# Persistent Post-Concussion Syndrome After Its Withdrawal from DSM-5 and ICD-11: Definitional Disagreement and the Neurocognitive Gap

**DOI:** 10.3390/jcm15208031

**Published:** 2026-10-16

**Authors:** Miguel A. Pappolla, Remi Nader, Felix Fang, Xiang Fang

**Affiliations:** 1 Department of Neurology, University of Texas Medical Branch, Galveston, TX 77555, USA; sxfang@utmb.edu; 2 Department of Neurosurgery, University of Texas Medical Branch, Galveston, TX 77555, USA; 3 Department of Physical Medicine and Rehabilitation, University of North Texas Health Science Center (UNTHSC), Fort Worth, TX 76104, USA; felix.fang@texasrehabhospital.com; 4 Texas Rehabilitation Hospital, Fort Worth, TX 76104, USA

**Keywords:** mild traumatic brain injury, concussion, post-concussion syndrome, persistent post-concussion symptoms, diagnostic criteria, DSM-5, ICD-11, neurocognitive disorder, vestibular, oculomotor, central sensitization

## Abstract

A substantial minority of patients do not recover fully after mild traumatic brain injury. Persistent post-concussion syndrome includes both cognitive and non-cognitive manifestations, yet DSM-5 and ICD-11 withdrew the syndrome as a distinct diagnosis and reorganized its persistent sequelae exclusively within neurocognitive categories. While this reorganization addresses the cognitive component of the syndrome, it provides no single category for its non-cognitive consequences, including vestibular, oculomotor, headache-related, autonomic, sleep, and pain-related manifestations. In some patients, these may be the most disabling consequences of the injury. This narrative review examines the resulting diagnostic gap and other continuing disagreements. Published criteria differ in symptom count, duration, and other requirements, yielding an unacceptably wide range of prevalence estimates, from 11.4% to 38.7%. We review the current status of this syndrome and propose an approach that separates diagnosis from attribution. Finally, we discuss the interactions between cognitive and non-cognitive consequences through shared neural substrates, so abnormalities in one domain may influence function in another. Because cognitive and non-cognitive manifestations may dissociate, a patient may have normal or minimal findings in one domain and functionally significant abnormalities in the other; therefore, evaluation confined to one domain alone (as reflected in the new classifications) cannot exclude the syndrome.

## 1. Introduction

Most people recover from a mild traumatic brain injury (mTBI). Within days or weeks, the headache resolves, the fog lifts, sleep returns, and life resumes. For a substantial minority, it does not happen that way. Months or years after an injury that the hospital record may describe as minor, these patients continue to report daily headache, dizziness in visually busy environments, intolerance of screens and noise, difficulty with memory and concentration, unrefreshing sleep, neck and shoulder pain without an imaging correlate, and irritability that is new to them and to their families. Many are unable to return to their previous work.

The existence of this clinical picture is not in question. It has been documented consistently in civilian, athletic, and military populations, in prospective cohorts and in clinical series, by investigators who hold differing views of its mechanisms [1,2,3]. What remains contested is the size of the affected minority. A scoping review of 45 studies reported that approximately half of individuals with a single mTBI demonstrated long-term cognitive impairment, with 1963 of 3593 participants meeting the authors’ criterion, stratified as 50% of children and 58% of adults [4]. A multinational review estimates that post-concussion symptoms persist in 10 to 25% of mTBI patients and reports a broader range of symptom prevalence, from 11 to 82%, depending on the diagnostic criteria, population, and timing of assessment [1]. The scoping review itself drew a published rebuttal arguing that its definition of impairment was idiosyncratic, in particular that a statistically significant group difference on a single cognitive test had been treated as establishing impairment in every injured participant, and concluding on re-review of the same studies that a single mTBI is not associated with a high incidence of chronic cognitive impairment [5]. The exchange repays attention because of what the two sides were and were not arguing about. Neither disputed that some patients have persisting impairment. What they disputed was what counts as impairment and how it should be ascertained, which is a question of definition. The truth lies somewhere in between.

That is the recurring theme of this review. The diagnostic apparatus built around persisting symptoms after concussion is genuinely unsettled. Several formal criteria sets exist and disagree with one another about symptom count, duration, and whether objectively demonstrated cognitive impairment is required. Applied to the same patients, they identify substantially different groups. Both current international diagnostic manuals have withdrawn the category and replaced it with a narrower one covering only the cognitive component. A reader encountering this literature for the first time can easily conclude that the condition itself is in doubt, and clinicians and evaluators sometimes argue as though it were.

We think that conclusion is mistaken, and this review is organized to show why. Disagreement about how to define a condition is the ordinary situation for a clinical syndrome that lacks a confirmatory test; it has characterized migraine, fibromyalgia, irritable bowel syndrome, and chronic fatigue syndrome at various points without implying that the patients were well. Criteria sets are instruments for identifying a population consistently, and when several instruments disagree, the finding concerns the instruments. Keeping the entity and its definition separate has three practical consequences that structure what follows: any statement about whether a patient meets criteria should identify which criteria; the withdrawal of a diagnosis from a manual is a fact about nomenclature rather than pathology; and the diagnosis of the syndrome and its attribution to an injury are separate determinations requiring separate reasoning.

Our objectives are to quantify the extent of the definitional disagreement; to set out what each of the major criteria sets requires; to examine what DSM-5 and ICD-11 did, why, and what gap their reorganization created; to propose an operational approach including causation analysis; and to argue, from the interaction between cognitive and non-cognitive consequences, that the evaluation of this syndrome cannot be confined to cognitive or to non-cognitive impairment alone, or to a single discipline.

## 2. Methods

This is a narrative review. We searched PubMed and Google Scholar for English-language publications from database inception through August 2026, with no lower date limit, using combinations of the terms post-concussion syndrome, persistent post-concussion symptoms, persisting symptoms after concussion, postconcussional disorder, mild traumatic brain injury, diagnostic criteria, DSM-5, ICD-11, neurocognitive disorder, retrospective diagnosis, vestibular, oculomotor, and central sensitization. Additional targeted searches, made during revision in October 2026, addressed base rates of post-concussion-like symptoms in uninjured populations, expectation and misattribution, and symptom and performance validity. We prioritized consensus statements, formal criteria sets, studies that applied multiple criteria sets to a single cohort, and reviews addressing the reclassification of the syndrome. Primary sources were retrieved for all criteria sets discussed and for all quantitative claims. Because the review is narrative, no formal quality assessment or meta-analysis was performed, and the selection of sources reflects the authors’ judgment of relevance.

## 3. The Scale of the Definitional Disagreement

A clinician who asks what the diagnostic criteria are for persistent post-concussion syndrome will find several answers in conflict, and the extent of the conflict is greater than most assume.

Rose, Fischer and Heyer opened their study of the question with the statement that a standard definition of post-concussion syndrome does not exist [6]. Surveying 597 physician members of the American College of Sports Medicine, they found substantial disagreement on the most basic parameter, how long symptoms must persist: 26.6% required less than two weeks, 20.4% two weeks to one month, 33% one to three months, and 11.1% more than three months [6]. These physicians were disagreeing by an order of magnitude about when to call the condition persistent. They were not disagreeing about whether their patients had it.

Voormolen and colleagues quantified the consequence. In 731 patients assessed six months after mTBI, applying divergent classification methods to the same Rivermead Post-Concussion Symptoms Questionnaire data produced prevalence estimates from 11.4% to 38.7% [2]. The methods differed only in how the same responses were read: a mapping to ICD-10 and DSM-IV criteria, the Rivermead total score, the RPQ-3 subscale, and a three-factor model, each at two severity cutoffs [2]. Nothing about the patients changed. Every method identified a substantial group of impaired patients, and what they disagreed about was where to draw the line around that group, with the most inclusive method identifying a group more than three times the size of the most restrictive.

Two conclusions follow: A statement that a patient does or does not have persistent post-concussion syndrome carries little meaning unless the definition being applied is identified. And a disagreement of this magnitude is a serious methodological problem about criteria; none of these studies found that the patients were well, and none was designed to ask.

## 4. What the Formal Criteria Require 

### 4.1. ICD-10 Clinical Criteria

The ICD-10 clinical criteria for postconcussional syndrome (F07.2) require a history of head trauma together with three or more of eight symptoms: headache, dizziness, fatigue, irritability, insomnia, difficulty concentrating, memory impairment, and reduced tolerance to stress, emotional excitement, or alcohol [7] (Table 1). They specify a symptom count and no duration after which the syndrome becomes persistent, an omission that forced researchers to invent their own operational durations and contributed to the heterogeneity above [8]. The ICD-10 clinical criteria are distinct from the separate and stricter ICD-10 research criteria, and the two are frequently conflated.

### 4.2. DSM-IV Postconcussional Disorder

DSM-IV proposed postconcussional disorder as a provisional diagnosis for further study. Its criteria required a history of head trauma causing significant cerebral concussion; evidence from neuropsychological testing or quantified cognitive assessment of difficulty in attention or memory; three or more of eight symptoms beginning shortly after trauma and persisting at least three months, drawn from fatigability, disordered sleep, headache, vertigo or dizziness, irritability or aggression, anxiety or depression or affective lability, personality change, and apathy; onset following trauma or substantial worsening of pre-existing symptoms; significant social or occupational impairment; and exclusion of dementia due to head trauma and other better explanations [7]. The distinctive requirement is the second. DSM-IV made objectively demonstrated cognitive impairment mandatory, so that a patient with disabling post-traumatic headache, vestibular dysfunction, photophobia, and mood disturbance, but intact cognitive testing, did not meet the criteria. Whether that requirement was well conceived is the question Section 9 takes up.

### 4.3. The Lagacé-Legendre Expert Consensus Definition

The clearest modern explicit definition comes from a Delphi consensus reported by Lagacé-Legendre and colleagues [8]. Persistent post-concussion symptoms require an established mTBI, since this is a post-injury diagnosis; at least one new post-concussion symptom, the panel having declined to require a minimum number; symptom onset within hours of the injury; that the symptom cannot be attributed to a pre-existing condition and is not better accounted for by another mental disorder; that at least one symptom is, in the panel’s language, still present every day 3 months after the trauma; and that it has an impact on at least one sphere of a person’s life [8].

The limitations should be stated as clearly as the criteria. This is an expert consensus rather than a validated instrument or an adopted professional-society standard, and the authors stated that future research is needed to evaluate its reliability and validity [8]. The panel did not reach agreement that three or more symptoms should be required, that any Rivermead score should establish the diagnosis, that any inventory or test should serve as a mandatory cutoff, that any particular initial severity was necessary, or that worsening of a pre-existing symptom qualifies as a new symptom [8]. One consequence is that the Rivermead questionnaire, valuable for documenting symptom type, burden, and change, is not supported by the literature as a diagnostic instrument, and no accepted cutoff diagnoses this condition.

### 4.4. The Four-Week Threshold

The 2023 Amsterdam consensus statement on concussion in sport uses a shorter threshold, conventionally more than four weeks, under the terminology of persisting symptoms after concussion [9]. It is a current and relevant consensus document, but the threshold is primarily a temporal and management designation rather than a causal diagnostic definition; it identifies when continuing symptoms warrant detailed multimodal evaluation and allows that such symptoms may be pre-existing, concussion-related, or both. A separate four-week figure circulates from the historical ICD-10 research criteria, where it referred to the permissible interval between injury and symptom onset rather than to persistence, and where loss of consciousness was also required, inconsistently with contemporary practice. Neither four-week figure is the current standard for persistent post-concussion syndrome.

**Table 1:** Principal definitions of persistent post-concussion syndrome compared.

Definition	Symptom Count	Duration Required	Objective Cognitive Impairment Required	Status
ICD-10 clinical criteria (F07.2)	Three or more of eight	Not specified	No	In clinical use during transition to ICD-11 [7]
DSM-IV post-concussional disorder	Three or more of eight	At least three months	Yes, mandatory	Provisional; superseded [7]
Lagacé-Legendre expert consensus	At least one; no minimum agreed	Daily at three months	No	Expert Delphi consensus, not a society standard [8]
Amsterdam 2022, persisting symptoms after concussion	Not specified	Conventionally more than four weeks	No	Management designation rather than causal definition [9]
ICD-11	Category withdrawn	Not applicable	Applies to successor category	Term retained only as an index entry under mild neurocognitive disorder (6D71) [3,10]
DSM-5	Category withdrawn	Not applicable	Applies to successor category	Replaced by major or mild neurocognitive disorder due to TBI [11]

The columns that differ most are symptom count and duration, which is why prevalence estimates in a single cohort vary more than threefold [2].

## 5. The Withdrawal from DSM-5 and ICD-11

Neither current international diagnostic manual contains post-concussion syndrome as a category. DSM-5 withdrew postconcussional disorder and folded the persistent cognitive sequelae of TBI into major or mild neurocognitive disorder due to traumatic brain injury [11,12]. ICD-11 made the corresponding change: the category that appeared in ICD-10 as F07.2 has no direct successor, and the persistent sequelae are subsumed under mild neurocognitive disorder, which may be specified as secondary to trauma [3]. The term itself is retained only as an index entry under that category [10]. The two revisions should be read as one decision. Both withdrew a symptom-cluster category defined chiefly by subjective complaints and replaced it with a neurocognitive category defined by objectively demonstrable cognitive impairment.

One proposed explanation concerns the inconsistency of the criteria, rather than a finding that the patients were well. Boake, McCauley and colleagues applied DSM-IV and ICD-10 criteria to the same patients three months after injury, in 178 adults with mild to moderate TBI and 104 with extracranial trauma. Prevalence was 64% under ICD-10 and 11% under DSM-IV, a nearly sixfold difference produced by the choice of criteria set alone [7]. Specificity was limited because the criteria were often satisfied by patients with extracranial trauma and no head injury, and the authors concluded that further refinement was needed before routine use [7]. A companion analysis of the same 178 patients found substantial agreement for symptom criteria (κ = 0.73) but only slight agreement for overall diagnoses (κ = 0.13), with kappa measuring agreement beyond that expected by chance [13]. Clark and colleagues suggest that inconsistency between the criteria may have contributed to the withdrawal from both manuals [3]. What the manuals withdrew was a category that two established criteria sets could not apply consistently to the same patients. The finding concerned the criteria rather than the patients.

Three consequences follow, and they are easily misread in either direction.

First, the withdrawal was a reorganization rather than a repudiation. Neither manual concluded that patients with persisting symptoms are not ill; each relocated the cognitive component to a category defined by demonstrable impairment. A coherent description of a patient therefore uses both frameworks where both apply, recording the post-concussive syndrome in the terms of the classification that recognizes it and mild neurocognitive disorder due to TBI where the cognitive criteria are satisfied. These are two nomenclatures describing one patient.

Second, the successor categories inherited much of the problem they were meant to solve. Clark and colleagues, having described the move to the neurocognitive categories, observe that the criteria for neurocognitive disorder continue to lack diagnostic precision and focus on non-specific symptoms [3]. Reorganization has not produced the specificity its rationale required, which argues for developing better objective methods rather than for narrowing the population recognized as injured.

Third, and least discussed, the reorganization created a gap. Both successor categories are neurocognitive. Each captures the cognitive consequences of the injury and, by construction, captures none of the vestibular, oculomotor, headache, autonomic, sleep, or chronic central neuropathic or nociplastic pain consequences. The withdrawal has been criticized directly by Auxéméry and Gayraud, who observe that it coincided with the elevation of post-traumatic stress disorder to its own category of trauma-related disorders, to which post-concussion syndrome could legitimately have been added, and who maintain that the syndrome remains a clinical-biological reality [14]. The gap has also been acknowledged in passing by authors otherwise sympathetic to the reorganization: Iverson notes that the DSM-5 category does not include post-concussion symptoms, being based instead on objective evidence of cognitive decline [15], and Clark and colleagues observe that the use of PCS as a diagnosis remains pervasive despite its removal from both manuals [3]. What has not, to our knowledge, been stated is the specific population the reorganization left without a category of its own. ICD-11 itself records the withdrawal. Postconcussional syndrome no longer has a category, a code, or diagnostic requirements of its own, and the term is retained only as an index entry under mild neurocognitive disorder (6D71) [10]. An index entry is a pointer that tells the coder where a term has been filed. It is not a code and does not restore the category. Its effect is that a record of postconcussional syndrome is coded as a cognitive disorder. The category it points to requires objective evidence of cognitive impairment and describes that impairment as not severe enough to interfere significantly with functioning [10]. A patient with intact cognition does not meet it.

The individual non-cognitive manifestations can still be coded one at a time, for example as persistent headache associated with traumatic injury to the head, as a vestibular disorder, or as chronic post-traumatic pain [10]. These codes do not replace what was withdrawn, for three reasons. Each records a single manifestation, so the diagnosis placed is headache or pain and not a post-concussive syndrome. Some are linked to trauma individually, but nothing connects the manifestations to one another or records their common onset in the acute period and their continuous course from the injury. In addition, *none of these codes require that the symptom has altered the patient’s function, which the syndrome definitions do require* [7,8]. ICD-11 allows function to be recorded through optional codes for pain-related interference and a supplementary section for functioning assessment, but it is not a condition of these diagnoses [10]. A patient whose predominant disability is vestibular and oculomotor, with intact cognition, therefore has in DSM-5 and ICD-11 no single category that recognizes the condition as a consequence of the injury, despite being demonstrably impaired by it.

In the authors’ clinical experience, which is drawn from a referral population with persistent symptoms, such patients make up the largest proportion of post-concussion patients. Published figures are consistent with that experience. Meta-analytic evidence indicates that performance on standard cognitive tests recovers within about 90 days of injury in most patients [16], yet 53% of 1154 patients with mTBI seen at level I trauma centers still reported functional limitations at 12 months, compared with 38% of orthopedic trauma controls [17]. In preliminary data from 236 patients at a concussion specialty clinic, migraine (26%) and anxiety or mood (24%) were the most common clinical profiles, and vestibular and ocular profiles together accounted for a further 35% [18]. New or worse headaches were present in 58% of patients one year after mTBI [19], and a systematic review estimated the prevalence of chronic pain among civilians with mild TBI at 75.3% [20]. In a controlled follow-up of mild closed head injury, patients had no cognitive impairment later in the first year but retained deficits in eye and arm motor function and elevated symptom levels, and the motor deficits remained measurable at 12 months [21].

The disabling weight of a given impairment also depends on what the person’s work and daily life demand of the affected system, a principle set out in the International Classification of Functioning, Disability and Health [22]. A scaffold worker, a ballet dancer, or a circus performer may be unable to work because of a balance or oculomotor deficit that an office worker would tolerate. A physicist may be disabled by mild cognitive slowing that would not limit manual work. Disability therefore cannot be read from the cognitive profile alone, and these observations argue for retaining a clinical construct alongside the neurocognitive categories rather than in place of them.

Practice has responded to the withdrawal, as clinical practice usually responds to nomenclature that does not fit what clinicians see. The terminology has continued to evolve, from post-concussion syndrome to persistent post-concussion symptoms to persisting symptoms after concussion, and the entity has continued to be recognized, treated, and studied under each name. ICD-10 also remains in clinical use in much of the world during a transition proceeding at different rates in different jurisdictions.

A related development should be acknowledged. A body of work has reframed persisting symptoms in biopsychosocial terms, emphasizing pre-injury, psychological, social, and environmental contributions to symptom maintenance [3]. That literature makes a legitimate point: attributing every continuing complaint to the concussion alone is not defensible, and treatment directed at maintaining factors is often more productive than treatment directed at the injury. What it does not establish is that the injury made no contribution. The more complete account holds both: the injury occurred, it initiated the syndrome, and its maintenance is multifactorial.

## 6. A Proposed Operational Approach

Given the state of the definitions, we do not propose a further criteria set. We propose a sequence for applying the established ones, and we identify the two elements that reflect the authors’ clinical judgment. The injury is established under the criteria of the American Congress of Rehabilitation Medicine (ACRM) [23]. The syndrome is then defined by the elements of the Lagacé-Legendre consensus: onset in the acute period, daily persistence at three months, functional consequence, and exclusion of better explanations [8]. The two elements that are our own are a symptom-count convention (Section 6.3) and a causation analysis conducted separately from diagnosis (Section 6.4). Table 2 summarizes the sequence and the source of each element.

### 6.1. Established Mild Traumatic Brain Injury

The diagnosis presupposes the injury, which should be established under contemporary diagnostic criteria, most authoritatively the 2023 ACRM criteria, which permit diagnosis through any of three routes: an acute clinical sign; two or more qualifying acute symptoms together with an objective examination or laboratory finding; or trauma-related abnormality on structural neuroimaging [23]. Establishing the injury does not, however, mean that it was documented at the time, and the two are routinely confused.

Most patients with persistent symptoms are first evaluated for brain injury weeks or months after the event, and for many the acute record is silent. In a prospective emergency department series, 56% of patients meeting criteria for mTBI received no such diagnosis in their record [24]. The reasons are structural. Acute care is organized around injuries that threaten life; patients with multiple injuries are examined for fractures, bleeding, and visceral trauma, and the brief confusion that would have signaled a concussion goes unelicited. Analgesia compounds this: in a level I trauma center series of 3042 patients exposed to a mechanism of mTBI, 65.1% had at least one factor complicating identification, most commonly opioids administered in the emergency department, given to 43.7% [25]. In the polytrauma population the absence of acute documentation is the expected finding, and it is not evidence that the injury did not occur.

For these patients the injury is established retrospectively, and retrospective establishment is the recognized method rather than a compromise. Walker and colleagues describe the clinical interview as the existing gold standard for diagnosing a suspected previous mTBI [26], validated structured interviews exist for the task [26,27], and the ACRM criteria state that mechanism, clinical signs, and acute symptoms may all be established retrospectively through detailed history, with attention to recall bias, response bias, and confounders [23]. The evidentiary hierarchy for such reconstruction runs from direct observation through collateral report and contemporaneous records to the patient’s own account [23].

What retrospective diagnosis reconstructs is the injury: mechanism, acute signs, and acute symptoms, with exclusion of alternatives. That differs from inferring an injury from the persistence of present symptoms alone, and the distinction is the one that matters. A patient whose record shows a high-energy collision, whose collateral history describes confusion at the scene, whose own account places headache and dizziness within hours, and in whom no other condition accounts for the picture, has an injury established by evidence about the injury. A patient whose only evidence is that headache and poor concentration are present now has not, because those symptoms are nonspecific and their presence at three months does not by itself locate an injury at time zero.

### 6.2. Temporal Onset, Persistence, Functional Consequence, and Exclusion

Symptoms should have appeared in the acute period, consistent with the Lagacé-Legendre requirement of onset within hours [8] and the ACRM window of generally less than 72 h for physical, cognitive, and emotional symptoms [23]. Persistence at three months is the more conservative of the current thresholds; four weeks is a reasonable point to intensify evaluation and treatment [9], and three months is the more defensible point to apply the label. Symptoms must have an impact on at least one sphere of life [8]. And the presentation must not be better accounted for by a pre-existing condition or another disorder.

### 6.3. Symptom Count

In the authors’ clinical practice, four or more symptoms from the 13-item subscale of the Rivermead questionnaire (RPQ-13) are required before the diagnosis is applied. This is the authors’ preference, adopted to increase specificity in a condition whose individual symptoms are common in uninjured populations [28,29]. It is not an established requirement, and conventional practice does not require it: the ICD-10 convention is three symptoms [7], and the Lagacé-Legendre definition sets no minimum number [8]. Fewer symptoms suffice when they are severe enough to produce significant functional impairment. This applies in particular to patients whose principal manifestations, such as headache and dizziness, are not among the RPQ-13 items. The three-symptom convention originates in ICD-10 and DSM-IV [7] and was operationalized on the full sixteen-item Rivermead by Ingebrigtsen and colleagues, who found that 62% of patients with minor head injury reported at least one symptom at three months while 40% met a three-symptom threshold [30]. Two cautions apply. That operationalization used the full instrument rather than the RPQ-13 subscale, and the RPQ-3 and RPQ-13 division derives from Rasch analysis by Eyres and colleagues as a severity scaling rather than a diagnostic checklist [31]. A symptom count applied to the Rivermead, including the authors’ own, is therefore a convention applied to that list, not a property of the instrument.

The exception matters as much as the threshold. Counting symptoms is a proxy for burden, and where burden can be assessed directly, the proxy should yield to it.

### 6.4. Causation

The elements above establish that a patient has the syndrome. They do not establish that the injury caused it, and the two questions are distinct. Clinical causation determination can follow three steps, adapted here from a framework developed for spinal disk injury [32]: a temporal relationship consistent with the mechanism; biological plausibility of the injury mechanism; and the absence of a better alternative explanation. Each takes a particular form here.

The temporal question has two parts. The first concerns onset in the acute period. The second concerns continuity: symptoms attributed to the injury should be traceable from the acute period to the present. A complaint first appearing months after injury raises a different causal question from one present since the first days. Delayed onset, meaning a manifestation genuinely absent acutely, requires a mechanism explaining the delay. Delayed recognition, meaning a manifestation present from the beginning and noticed only when circumstances demanded the impaired function, is common and causally unproblematic once the history is reconstructed. Distinguishing the two requires a detailed account of the first days rather than acceptance of the date of first complaint, and should be done symptom by symptom.

The injury must have involved a transfer of mechanical energy capable of producing brain injury [23]. Two qualifications keep this step accurate. No validated force threshold distinguishes injurious from non-injurious mechanisms in mTBI, and common proxies are unreliable: vehicle damage correlates poorly with occupant injury, airbag deployment is calibrated to crash dynamics rather than head injury risk, and absence of direct head impact does not exclude a mechanism. At the same time, magnitude is not irrelevant. The ACRM criteria adopt a probabilistic framing in which a witnessed hard blow creates high pre-test odds that lower the additional evidence required [23]; the corollary is that a trivial mechanism raises the evidentiary requirement.

The third step corresponds to the ACRM’s Criterion 6 and is more demanding at three months than at three days, because time introduces explanations unavailable acutely: medication-overuse headache develops from treatment rather than injury, deconditioning follows activity restriction, and sleep disruption, mood disturbance, and chronic pain each degrade cognition independently. Two principles govern. Coexistence is not exclusion: the question is whether an alternative accounts for the presentation better than the injury does, measured against the patient’s own premorbid function. And causation of the syndrome and causation of each symptom are different questions: the injury may be the initiating event while a given symptom is multifactorial, and the defensible formulation identifies the injury as initiating and describes maintaining factors explicitly.

### 6.5. Validation

The approach has not been validated, and it is offered as a clinical framework rather than as a criteria set. It could be tested in three ways. Inter-rater agreement could be measured among clinicians applying it to the same cases. It could be applied alongside the ICD-10, DSM-IV, and Lagacé-Legendre definitions in a single prospective cohort that includes orthopedic trauma controls, as was done for earlier criteria [2,7], to compare prevalence, agreement, and specificity. Its criterion validity could be examined against functional outcome and against objective vestibular, oculomotor, and cognitive findings.

**Table 2:** Sequence for applying established criteria to persistent post-concussion syndrome, with the source of each element.

Step	Requirement	Source
1. Injury established	Mild TBI established under the ACRM criteria, by retrospective reconstruction where the acute record is silent	Established: ACRM [23]; structured interview methods [26,27]
2. Acute onset	Symptoms began in the acute period	Established: Lagacé-Legendre (within hours) [8]; ACRM (generally within 72 h) [23]
3. Persistence	At least one symptom present daily at three months	Established: Lagacé-Legendre [8]. Amsterdam applies the term persisting symptoms after four weeks [9]; here that is the point to intensify evaluation (authors’ judgment)
4. Functional consequence	Impact on at least one sphere of life	Established: Lagacé-Legendre [8]
5. Exclusion	Not better accounted for by a pre-existing condition or another disorder	Established: Lagacé-Legendre [8]; ACRM [23]
6. Symptom count	Four or more RPQ-13 symptoms; fewer suffice when severe enough to produce significant functional impairment	Authors’ preference, adopted to increase specificity; not an established requirement. ICD-10 and DSM-IV convention: three symptoms [7,30]; Lagacé-Legendre: no minimum [8]
7. Causation, analyzed separately from diagnosis	Temporal relationship (acute onset and continuity), biological plausibility of the mechanism, and absence of a better alternative explanation	Authors’ judgment, adapted from Freeman and colleagues [32] and consistent with the ACRM criteria [23]

ACRM, American Congress of Rehabilitation Medicine; RPQ-13, 13-item subscale of the Rivermead Post-Concussion Symptoms Questionnaire; TBI, traumatic brain injury.

## 7. The Clinical Syndrome: Cognitive and Non-Cognitive Consequences

The syndrome is conventionally described in symptom lists. It is more useful to organize it into cognitive and non-cognitive impairment, and within the latter by system, because these map to different anatomy, different assessment methods, and different treatments, and because the division into cognitive and non-cognitive consequences organizes the argument of Section 8 and Section 9.

Cognitive impairment after mTBI is typically selective rather than global, affecting attention, memory, executive function, and processing speed in varying combinations. Two features recur: Standard cognitive testing is a resting measure, administered one task at a time in quiet conditions, and it systematically under-detects the divided-attention failure that defines many patients’ real-world impairment. And a normal bedside screen, including a Mini-Mental State Examination score of 30, does not exclude impairment after mTBI. The instrument was developed to grade cognitive state in patients with dementia and other major neuropsychiatric disorders [33], and its sensitivity after mTBI is low, reported as 0.43 to 0.58 [34].

The non-cognitive consequences are frequently the more prominent and, in our experience, often the more disabling. Evidence-based classifications of concussion recognize headache or migraine, vestibular, ocular-motor, and anxiety or mood subtypes alongside the cognitive one [35], and in preliminary data from a concussion specialty clinic these non-cognitive profiles were the majority of those identified [18]. Post-traumatic headache most often has a migraine phenotype [19] and organizes a patient’s day around avoidance; medication overuse is a treatable complication and is strongly associated with persistence of the headache [36]. Dizziness and imbalance may be vestibular, oculomotor, cervicogenic, or autonomic in origin, and the treatment differs with the mechanism [37]. Oculomotor dysfunction, including convergence insufficiency and impaired pursuit and saccades, produces reading and screen intolerance with normal visual acuity, because the problem lies in the control of eye movement rather than in the resolution of the image [38,39]. Autonomic dysregulation produces orthostatic intolerance and exercise intolerance [37,40], which can be mistaken for anxiety or deconditioning. Disturbed sleep is among the most common complaints and worsens the other sequelae [41]. Central auditory processing impairment presents as difficulty following speech in noise that pure-tone audiometry does not explain [42]. Mood and behavioral change may be direct consequences of the injury, reactions to it, or both [1,3].

Chronic pain deserves specific attention because its mechanism has recently been clarified. Persistent cervical, shoulder, and back pain is common after the mechanisms that produce concussion. Patients may have nociceptive pain from tissue injury, neuropathic pain from injury to somatosensory pathways, and nociplastic pain arising from altered nociceptive processing without evidence of tissue or nerve damage, with the underlying mechanism commonly described as central sensitization. Our group addressed which pain type and mechanism predominates in a cohort with persistent post-concussion syndrome and chronic pain, and identified central sensitization as the principal (but not the only) mechanism, concluding that chronic pain in this population is likely driven by central nervous system pathology contributing both to the pain experience and to other post-concussive symptomatology [43]. That finding comes from a single cohort and requires replication. It is consistent with independent work showing cranial hyperalgesia and diminished endogenous pain modulation in patients with mTBI and chronic post-traumatic headache [44], and with an exploratory prospective study in which reduced pain inhibitory capacity one to two weeks after mTBI predicted persistent post-traumatic headache at four months [45]. Two implications follow: Patients with high central sensitization frequently report severe pain localized to discrete regions, leading clinicians to direct antinociceptive therapies and interventional procedures at those regions, which is frequently ineffective because it addresses a peripheral target for a centrally generated problem [43]. And because nociplastic pain arises from altered nociceptive processing rather than from ongoing tissue or nerve damage [46], imaging of the painful region often shows findings that do not account for the pain. A normal study is therefore compatible with the mechanism and is not evidence against it, although nociplastic pain may coexist with nociceptive and neuropathic sources that imaging does identify.

Two patients may carry the same diagnosis and share almost no clinical features. A research technician whose disability is cognitive slowing and reduced attentional endurance, with mild dizziness she barely notices, cannot do her job; a roofer whose cognition is essentially intact but who is unsteady on scaffolding with incoordination and cervical pain that prevents rapid head rotation cannot do the work. A neuropsychological evaluation would capture the first and characterize the second as unimpaired. That contrast is further discussed in Section 9.

## 8. The Interaction Between Cognitive and Non-Cognitive Impairment

The cognitive/non-cognitive division organizes assessment, and it misleads if taken to imply that the two categories operate independently. They do not. The systems interact; they share neural substrates, and a deficit measured in one may originate in another. Three consequences follow: a cognitive finding may be produced by a non-cognitive mechanism, treating a non-cognitive system may improve cognition, and the total functional burden exceeds the sum of individual deficits.

The clearest example is the relationship between oculomotor control and cognition, because the anatomy is shared rather than adjacent. Saccades, smooth pursuit, fixation stability, and gaze holding depend on frontal eye fields, dorsolateral prefrontal cortex, posterior parietal cortex, superior colliculus, brainstem, and cerebellum [38]. Those same frontoparietal networks subserve attention, working memory, and inhibitory control. The antisaccade task, in which a subject must suppress a reflexive glance toward a target and look away from it, is simultaneously an oculomotor measurement and a measurement of inhibitory control, and is used as both.

The functional consequences follow several routes. Visual information is acquired through eye movement, so inefficient saccades and unstable fixation degrade acquisition before any question of storage arises; a patient who performs poorly on visual memory testing may have a memory deficit, or may have acquired the material incompletely because the oculomotor system delivered it poorly, and the two are indistinguishable on the memory score. Reading places heavy demands on precisely metered saccades, stable fixation, and sustained convergence, so convergence insufficiency and pursuit abnormality produce reading intolerance and loss of place that present clinically as impaired concentration. Compensation consumes resources: a patient exerting effort to stabilize gaze has less attentional capacity for content, which is why oculomotor deficits become most apparent in visually complex environments and under multitasking demands. These are often not measured in standard neuropsychological assessments. Oculomotor abnormalities after mTBI correlate with cognitive slowing, attentional difficulty, and visual motion sensitivity for these reasons [39]. Network analysis of Rivermead data in two independent mTBI cohorts, one at six weeks and one ten years post-injury, found blurred vision among the most central symptoms in the network in both, alongside frustration and concentration difficulty, with its centrality stable across the decade [47]. A symptom that is central in a network is one whose activation propagates to others, which is what a shared-substrate account predicts.

Vestibular input contributes to spatial orientation and memory, and postural control draws on attentional resources, measurable as a dual-task cost [48] and presenting as cognitive fatigue in environments demanding simultaneous movement and thought. Chronic pain degrades attention, working memory, and processing speed, and where the pain is nociplastic, the relationship is closer than competition for attention, because the central pathology generating the pain is the same pathology contributing to other post-concussive symptoms [43]. Central auditory processing impairment increases listening effort. Sleep disturbance degrades every aspect of cognition and lowers pain threshold. Depression and anxiety impair concentration and processing speed and are themselves worsened by pain, poor sleep, and functional loss.

The interpretive implication is that a cognitive test score measures performance rather than mechanism. Impaired visual memory may reflect impaired encoding, impaired oculomotor acquisition, or both. Impaired sustained attention may reflect an attentional deficit, or the cost of compensating for unstable gaze or posture, or pain, or a night without sleep, or several at once. Determining which requires assessment of the other systems and cannot be done from cognitive testing alone. The therapeutic corollary is easily overlooked: correcting convergence insufficiency, treating vestibular dysfunction, resolving medication-overuse headache, treating sleep-disordered breathing, or addressing centrally mediated pain may each yield cognitive improvement without any cognitive intervention.

## 9. The Limits of Cognitive Testing Considered Alone

This section addresses a recurring source of diagnostic error, stated carefully because the point is easily overstated in ways that are themselves inaccurate.

Persistent post-concussion syndrome has cognitive and non-cognitive consequences, and the non-cognitive systems are assessed by methods and instruments entirely distinct from those used to assess cognition. Two limits follow, and they run in opposite directions. When an evaluation confined to cognitive impairment, or to a single non-cognitive system, is negative, it cannot exclude the syndrome, because the syndrome may reside entirely in what was not assessed. When it is positive, the finding may be attributed to the injury if the causation analysis of Section 6.4 is satisfied, but its mechanism cannot be established from that evaluation alone, because, as Section 8 shows, the systems interact and share substrate, and a deficit measured in one may originate in another. A single-system evaluation may therefore miss what it did not assess and misattribute the mechanism of what it did.

Of these two limits, the first is the one most often overlooked in practice, and it usually arises as follows. A neuropsychological evaluation is performed, cognitive testing is within normal limits or mildly abnormal, and the conclusion drawn is that the patient does not have a TBI, or does not have post-concussion syndrome, or has no residual injury-related impairment. The first two conclusions do not follow from the data. The third does not either, unless expressly limited to cognition.

The reason is scope rather than the competence of the evaluator. Neuropsychologists are the appropriate experts in cognitive assessment, and their instruments, normative data, and validity methodology are indispensable, particularly for detecting non-credible performance. We have set out elsewhere, with a clinical neuropsychologist as co-author, the distinction between cognitive assessment and neuropsychological assessment, the respective roles of neurologists and neuropsychologists in each, and the reason cognitive test results interpreted in isolation are readily misconstrued as comprehensive measures of brain function when non-cognitive neurological findings are not integrated with them [49]. The limitation has been stated most forcefully from within that discipline. Bigler and colleagues, in a four-part review in *Brain Injury*, observe that the absence of neuropsychological findings has been used to argue no residual neuropathological effects from mTBI, and conclude that traditional neuropsychological tests as standalone findings cannot address the complexity of detecting mTBI pathology, having been developed as omnibus measures for neurological and neuropsychiatric conditions generally and never designed for the subtle and multifaceted changes that follow mTBI [50,51]. Their training does not extend to vestibular examination, oculomotor and vestibulo-ocular assessment, headache phenotyping, evaluation of central and nociplastic pain, autonomic testing, or audiological assessment. A normal cognitive profile is evidence of normal cognition. It cannot exclude a condition whose principal manifestations in a given patient are vestibular, oculomotor, and pain-related.

Consider a patient with intractable migrainous post-traumatic headache, objectively abnormal optokinetic responses and smooth pursuit, convergence insufficiency, orthostatic intolerance, and cervical pain with features of central sensitization, whose cognitive testing in a sterile environment is almost normal. This patient has a severe post-concussive condition. A conclusion from the normal cognitive testing that no syndrome exists would extend to six areas of impairment, a finding that concerns one.

Three qualifications are necessary: The error is one of over-extension beyond the system assessed rather than of professional category; a neurologist who declares a patient cognitively intact on a bedside screen, without formal testing or validity measures, has committed the identical error in the opposite direction. A properly framed neuropsychological conclusion is legitimate and valuable: a finding that cognitive testing demonstrates no impairment in the cognitive domains assessed, with valid performance, is true and useful, and the difficulty arises only when a domain-limited finding is expressed as a global conclusion. And the principle cuts both ways: objectively abnormal vestibular or oculomotor findings do not establish cognitive impairment.

There is a second reason a normal cognitive evaluation cannot exclude impairment, and it applies even when the question is confined to cognition. Standard neuropsychological testing is administered one task at a time, in a quiet room, with the examiner controlling pace and eliminating distraction. It is, in the most precise sense, a resting measure. The impairment that many patients describe, and that their employers and families observe, arises under divided attention: holding one task while performing another, tracking conversation in noise, reading while monitoring the room, working under time pressure in a visually busy environment. Testing that never imposes that load will not detect a deficit that appears only under it. This is why so many patients, and their providers, first recognize the impairment on return to work or school rather than in the clinic.

An analogy makes the structure plain. A patient with chronic obstructive pulmonary disease may have entirely normal oxygen saturation sitting on a couch watching television and become breathless and desaturate on one flight of stairs. Nobody concludes from the couch measurement that the lungs are normal. The stairs are the real-world demand on the brain, and the quiet examination room is the couch.

The evidence for this is direct, although it is limited in scale. McDowell and colleagues found significantly greater performance decrements under dual-task conditions in 25 patients recovering from severe TBI than in controls; these patients also had deficits on other executive measures [52], and Cicerone found that patients with mTBI differed from controls only under dual-task demands that exceeded their available cognitive resources [53]. The effect persists: young adults with remote mTBI showed significantly greater decline in associative memory under divided attention than controls, with fatigue rising as performance fell, in the injured group only [54]. It extends to motor tasks, since patients with mTBI who did not differ from controls on symptom measures or neuropsychological testing nonetheless showed significantly greater dual-task cost in gait speed [48]. Functional imaging provided complementary evidence in male athletes aged 9 to 15 years: working memory performance was similar under single-task conditions, but increased load produced an altered pattern of neural activation in mTBI, uncovering impairment in youths who were not highly symptomatic and did not show neuropsychological dysfunction [55]. These studies are small and differ in population, injury severity, time since injury, and paradigm. Two of them, in severe TBI [52] and in pediatric athletes [55], are cited as convergent rather than direct evidence for adults with mTBI. The direction of the effect is nonetheless consistent across them, and it is supported by a meta-analysis of individual participant data in which athletes walked more slowly than controls under dual-task conditions, but not under single-task conditions, up to two months after concussion [56]. The consequence for interpretation is documented directly: most neuropsychological tests are weakly or entirely unrelated to return-to-work outcome after mTBI [57], and in a cohort of 1554 patients, cognitive performance at higher levels of functional outcome was similar across categories, so that any influence of cognition on overall function was markedly attenuated at the upper end [58].

A normal cognitive profile obtained at rest therefore establishes that the patient performs normally at rest. It does not establish that the patient performs normally under load, which is the condition under which persistent impairment is experienced and observed.

Three bodies of literature are often set against the account given here, and each makes a point that a complete evaluation must respect. First, post-concussion-like symptoms are common in people who have had no head injury. They are endorsed by healthy adults [28], by the general population, in which fatigue was reported by 49.9% of 11,759 respondents [29], and by patients with extracranial trauma [7]. Symptom report alone therefore cannot establish the syndrome. This is why the approach in Section 6 requires an established injury, onset in the acute period, continuity, functional consequence, and exclusion of alternatives, and does not rest on a symptom count. The symptoms that are common without injury are nonspecific and self-reported. Objective abnormalities are a different kind of evidence. In a controlled study, the eye-movement deficits of patients with post-concussion syndrome included measures beyond conscious control and were practically unaffected by group differences in depression and estimated intellectual ability [59]. Second, expectation and misattribution influence symptom reporting. People with no head injury can predict the symptoms expected after one, and injured patients tend to underestimate how often they had the same symptoms before the injury [60,61]. These processes belong to the multifactorial maintenance described in Section 5. They act chiefly on self-report and on effort-dependent performance, and they do not readily account for abnormalities in measures beyond conscious control. Third, symptom and performance validity assessment belongs in every evaluation, and particularly in medico-legal ones [62]. A failed validity indicator limits the interpretation of the scores it concerns and must be weighed with the rest of the evaluation. By itself it neither establishes nor excludes an injury. None of these considerations is an argument against the syndrome. Each is an argument for evaluating it with objective methods across more than one system.

The practical implication is that a complete evaluation addresses cognition, headache, vestibular and oculomotor function, pain including its central mechanisms, autonomic regulation, sleep, hearing, and mood, and that such an evaluation requires more than one discipline. Polinder and colleagues reached the same conclusion from a different starting point, arguing against a diagnosis of persistent post-concussion symptoms as a disorder in the absence of comprehensive multidimensional medical, neurological, psychiatric, and neuropsychological assessment [1]. Where an evaluation omits cognitive or non-cognitive impairment, or any system within the latter, its findings are silent on what was omitted rather than exclusionary of it.

## 10. Implications for Assessment and Management

The Rivermead questionnaire quantifies symptom type and burden across cognitive and non-cognitive impairment and is most informative on serial administration, but it identifies the symptom without identifying its mechanism, and dizziness scored as severe may be vestibular, oculomotor, cervicogenic, or autonomic with identical scores and different treatments. Objective assessment across the non-cognitive systems, through vestibular and oculomotor testing, posturography, autonomic assessment, and sleep study, complements neuropsychological testing with embedded validity measures for cognition.

Management follows the clinical profile rather than the label. Headache is treated on its phenotype, with attention to medication overuse. Vestibular dysfunction is treated with rehabilitation rather than rest. Oculomotor dysfunction is treated with oculomotor rehabilitation and, where indicated, optical correction. Cognitive impairment is treated with cognitive rehabilitation and graded return to load. Chronic pain requires an approach matched to its mechanism, since nociplastic pain responds poorly to peripherally directed treatment [43]. Autonomic dysregulation is treated with graded sub-symptom-threshold exercise. Sleep disturbance is treated directly and early, and mood disturbance in its own right. Because the systems interact, treatment of any of them may improve the others.

## 11. Limitations

This is a narrative rather than a systematic review, and source selection reflects the authors’ judgment. The approach in Section 6 has not been validated, and the symptom-count threshold described in Section 6.3 reflects the authors’ preference rather than validated criteria and is presented as such. The dual-task studies cited in Section 9 are small and heterogeneous, and the central sensitization finding cited in Section 7 comes from a single cohort. The argument from shared neural substrate in Section 8 rests on established oculomotor and attentional anatomy, on clinical correlation, and on the dual-task literature cited in Section 9; the specific claims regarding visual encoding and listening effort would benefit from dedicated prospective study in post-concussion populations. The clinical experience informing this review is drawn substantially from a referral population with persistent symptoms, and the proportion of all mTBI patients to whom its arguments apply is precisely the contested quantity discussed in Section 1.

## 12. Conclusions

Persistent post-concussion syndrome is a real clinical entity whose boundaries are contested. The formal criteria sets disagree about symptom count, duration, and the necessity of objective cognitive impairment, and applied to the same patients they identify groups differing more than threefold in size. Both DSM-5 and ICD-11 withdrew the category and replaced it with a neurocognitive one, a reorganization that may have reflected the inconsistency of the criteria rather than a finding about the patients, and one that left a gap. For patients whose persisting disability is vestibular, oculomotor, headache-related, or pain-related, with intact cognition, the manifestations can be coded only one at a time. No single category in either manual recognizes them as one condition arising from the injury, records their course from the acute period, or captures their effect on function. The only entry ICD-11 retains for the term postconcussional syndrome directs the coder to a cognitive category that these patients do not meet.

We propose an operational approach that establishes the injury by retrospective reconstruction where the acute record is silent; requires acute onset, persistence at three months, functional consequence, and exclusion of alternatives; treats symptom count as a proxy for burden; and analyzes causation in three steps separately from diagnosis. And we argue that because cognitive and non-cognitive consequences share neural substrate and interact, a cognitive test score measures performance without establishing mechanism, and because standard testing is a resting measure, a normal result establishes normal performance at rest rather than under the divided-attention load at which impairment is experienced. An evaluation confined to cognitive impairment alone, or to non-cognitive impairment alone, cannot exclude the syndrome when negative and cannot establish the mechanism of what it finds when positive. The evaluation of this condition requires assessment of both cognitive and non-cognitive impairment, and more than one discipline.

## Data Availability

No new data were created or analyzed in this study. Data sharing is not applicable to this article.
